# Impact of windstorm on a community of centipedes (Chilopoda) in a beech forest in Western Poland

**DOI:** 10.2478/s11756-018-0022-9

**Published:** 2018-03-26

**Authors:** Małgorzata Leśniewska, Filip Skwierczyński

**Affiliations:** Department of General Zoology, Adam Mickiewicz University, Umultowska 89, 61614 Poznań, Poland

**Keywords:** Windstorm, Chilopoda, Beech forest, Coarse woody debris

## Abstract

The study was carried out in the years 2016–2017, five years after a windstorm which destroyed 1/3 of the protected beech forest area in the west of Poland. The community of centipedes in the area affected by the windstorm was depleted in terms of the species richness, diversity, and population density. The dominance structures were shortened and the species composition was rebuilt. The areas that proved to be the richest in terms of species richness and diversity among the sites affected by the windstorm were the one where windfallen trees were left and the other where beech trees had been planted by humans. In total, the quantitative and qualitative samples collected four times throughout a year featured 608 specimens from 11 species of two centipede orders – Lithobiomorpha and Geophilomorpha. *Lithobius curtipes* and *L. forficatus* were found in all of the investigated areas. *L. pelidnus* and *L. piceus* were captured at control sites exclusively. Only one species –*L. erythrocephalus* was found solely at the damaged site. The most numerous and most frequently found species in the community were *L. curtipes*, *L. mutabilis*, and *Strigamia acuminata* respectively. Although windstorms are natural phenomena their consequences may lead to significant changes in the community of the investigated soil animals. The importance of coarse woody debris, significantly contributing to the improvement and maintenance of species richness and diversity of Chilopoda, has once again been confirmed.

## Introduction

Anthropogenic transformation of the environment has now reached an unprecedented scale causing natural disturbances which are projected to become increasingly intensified (Dale et al. 2001).

In Central Europe windstorms are some of the most serious natural disasters (Fischer et al. 2002). The consequence of their occurrence is usually the destruction of forest ecosystems: this may be minor, leading only to clearances in the forest structure or it can be extensive, such as windthrow, formed by hundreds of broken and fallen trees on a large area (Bouget and Duelli 2004).

Wind disturbances cause two important structural changes: the removal of canopy material and the deposition of that material on the forest floor or the complete removal of this material from the environment. This can lead to a sudden increase in organic matter and an increased amount of light reaching the forest floor. Over many years, decomposition of large amounts of organic matter can contribute to increased amounts of nutrients in the soil whilst blowdowns (overturned trees with roots) increase the heterogeneity of the environment (Coyle et al. 2017).

Windthrow leads to increased temperature fluctuations and the reduction of moisture in the ecosystem (Ulanova 2000; von Oheimb et al. 2007). These changes have a significant impact on the occurrence and distribution of organisms inhabiting the soil environment (Pickett and White 1985; Pontailler et al. 1997; Niemelä 1999; Pickett et al. 1999; Bengtsson et al. 2000; Chapin et al. 2002). The consequence of windthrow is the formation of new microhabitats conditioning or limiting the occurrence of many species of invertebrates (Bouget and Duelli 2004; Lindhe et al. 2004; Lindhe and Lindelow 2004). As far as soil arthropods in Central European forest ecosystems are concerned, this applies primarily to mites (Acari) and collembolans (Collembola), which constitute up to 98% of the total number of this group of animals (Błoszyk 1999; Kim and Jung 2008; Jung et al. 2010; Čuchta et al. 2012) and also other arthropods, including arachnids, myriapods, crustaceans, as well as larvae and adult forms of insects (Greenberg and Forrest 2003; Coyle et al. 2017).

The consequences of windthrow are changes in the composition of animal communities (usually depletion of the species pool) which plays an important role in ecosystem processes (e.g. nutrient dynamics and soil quality) (Coyle et al. 2017). This leads to changes in multi-trophic food webs and the stability of the ecosystem.

In the myriapodological literature, we find several works dealing with issues related to the influence of forest structure changes (caused by various reasons) on Chilopoda communities. Thus, the relationship between Chilopoda and the structure of beech forest stands of various ages was described by Grgič and Kos (2005), pointing out to the great role of age and structural heterogeneity of beech forests for the richness of Chilopoda communities. Tuf (2000) investigated communities of centipedes in the tree floodplain forests of various age. Stašiov and Svitok (2014) analyzed the influence of stand density on the structure of centipede (Chilopoda) community in the submountain beech forest. Urbanovičová et al. (2014) investigated the impact of differently managed windthrown forest stands in the High Tatra Mts on the activity of epigeic arthropods. The study also investigates centipedes as one of the groups, however without a more in-depth analysis of the species composition and of the community structures. All of these works therefore address a problem directly related to our work – how the forest structure affects the Chilopoda community.

The aim of our work is to determine the extent to which the windstorm has affected the centipede (Chilopoda) community by comparison of damaged and undamaged areas of the same forest.

## Methods

The study was conducted in the “Buczyna” reserve located in the western part of Poland (16°59″E, 52°40”N).

The studied area lies in the Gniezno Lake District in the central part of the Wielkopolska Lake District, in the moraine hills of the Baltic glaciation, of the Poznań stadial. This is where moraine clay is found, on which soils belonging to brown soils have formed. The area does not exceed 200 m above sea level, and it lies in the temperate climate zone, in the area of mutual penetration of sea and continental influences. The area is characterized by relatively low annual rainfall: 450–500 mm, the average annual temperature is 8.5 °C (Kondracki 2002).

The reserve covers a 15.71 ha fragment of a compact forest complex, where 100–150-year-old beech forests on brown soils predominate. The reserve encompasses the most valuable part of beech forests located at the eastern border of the natural range of beech trees in Central Europe. Generally, the structure of the forest stands is close to the natural condition, and the floristic composition is typical of fertile lowland beech forests, occasionally similar to oak-hornbeam forests. The largest area is occupied by a community *Melico-Fagetum* (with *Melica uniflora*) (Ferchmin 1980; Król and Szczepanik-Janyszek 1994). The reserve was included in the Natura 2000 network as a special habitat protection area (The Habitat Directive, Area code: PLH300056). It has long been one of the most interesting nature and forest sites in the vicinity of Poznań (Urbański 1930; Stolarski 1932; Wodziczko et al. 1938).

In July 2012, approximately 1/3 of the beech tree stands were destroyed in the aftermath of a windstorm. The only intervention involved human planting of beech trees at the border of the reserve.

We designated 6 study areas for investigation.

- Sites I and II – control sites – included parts of the forest that were not affected by the gale, with the surviving forest stand. It is a beech forest with rich undergrowth and thick brushwood of regenerating beech trees and with a small admixture of oak trees and a fragment of a forest made up of approximately 80 year old beech trees. In addition site II featured significant coarse woody debris.

The other four sites covered parts of the forest destroyed by the windstorm.

Sites III and IV (regenerating):

- Site III – with natural beech brushwood reaching the height of approx. 3 m and rich grassy undergrowth;
- Site IV – with human planting of beech trees (after the windstorm) where surviving, approx. 60 year old oak trees; beech and larch trees are also found.

**Fig. 1:**
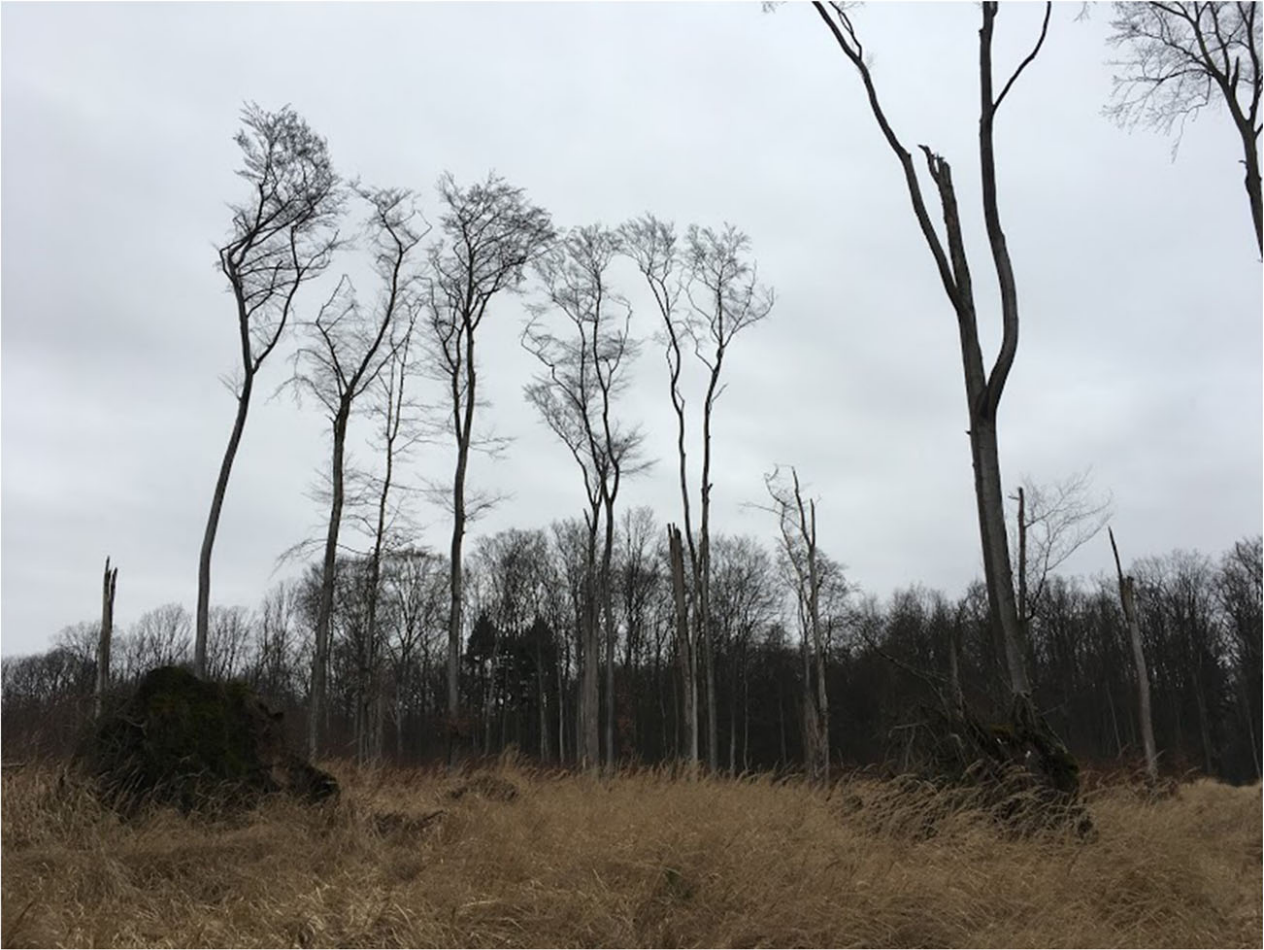
Site V

**Fig. 2:**
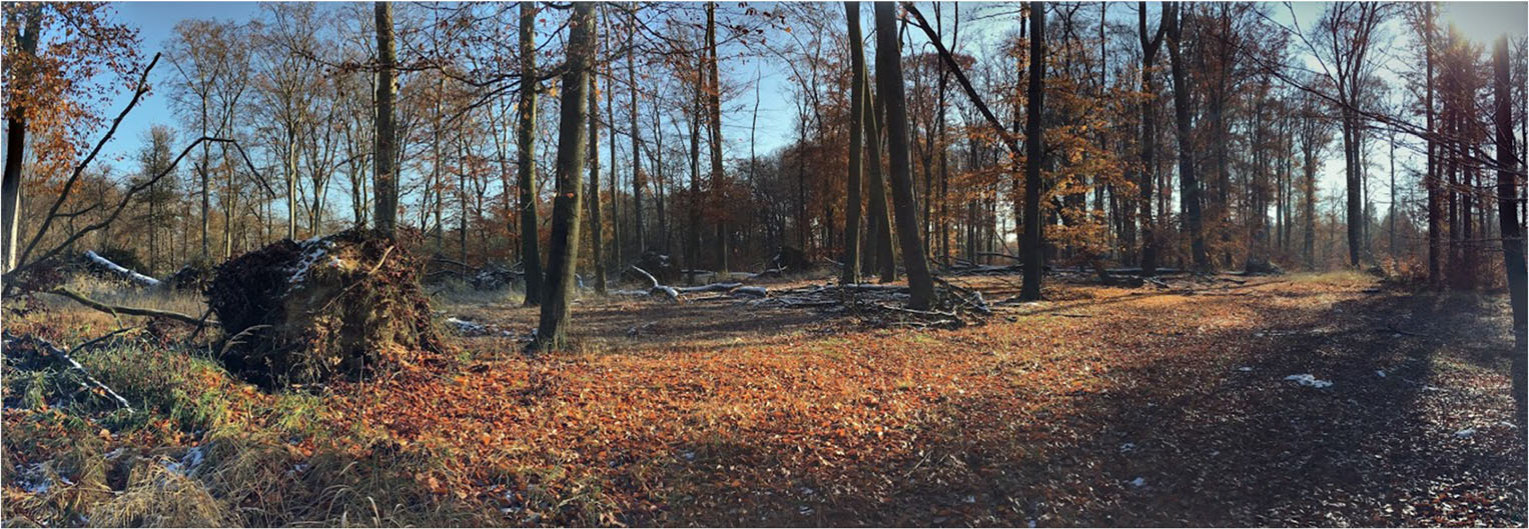
Site VI

Sites V (Fig. 1) and VI (Fig. 2) (forest areas with the greatest damage):

- Site V – this is windthrow with rich undergrowth (mainly dominated by *Calamagrostris epigeios*) featuring single standing beech trees;
- Site VI – windthrow with poor undergrowth, a few beech trees and numerous lying beech logs and coarse woody debris.

The quantitative material was collected four times: in spring (4 June 2017), in the summer (8 July 2016), in the fall (12 November 2016), and in winter (26 February 2017) (Table 1). The air temperature on the days of material collection was as follows: 4 June 2017 – 18 °C; 8 July 2016 – 21 °C; 12 November 2016 – -1 °C (no snow cover); 26 February 2017 – 6 °C.

**Table 1:** The number and area of quantitative samples and the percentage of samples with centipedes (CE%)

Site/Date	08.07.16		12.11.16		26.02.17		04.06.17		Total	
	All	CE%	All	CE%	All	CE%	All	CE%	All	CE%
I	8 (0.5 m^2^)	100.0	8 (0.5 m^2^)	87.5	8 (0.5 m^2^)	75.0	8 (0.5 m^2^)	62.5	32 (2.0 m^2^)	81.3
II	8 (0.5 m^2^)	100.0	8 (0.5 m^2^)	100.0	8 (0.5 m^2^)	75.0	8 (0.5 m^2^)	87.5	32 (2.0 m^2^)	90.6
III	8 (0.5 m^2^)	62.5	8 (0.5 m^2^)	12.5	8 (0.5 m^2^)	0	8 (0.5 m^2^)	50.0	32 (2.0 m^2^)	31.3
IV	8 (0.5 m^2^)	50.0	8 (0.5 m^2^)	50.0	8 (0.5 m^2^)	12.5	8 (0.5 m^2^)	50.0	32 (2.0 m^2^)	40.6
V	–	–	8 (0.5 m^2^)	37.5	8 (0.5 m^2^)	0	–	–	16 (1.0 m^2^)	18.7
VI	8 (0.5 m^2^)	75.0	8 (0.5 m^2^)	62.5	8 (0.5 m^2^)	37.5	8 (0.5 m^2^)	62.5	32 (2.0 m^2^)	59.4
Total	40 (2.5 m^2^)	75.0	48 (3.0 m^2^)	58.3	48 (3.0 m^2^)	33.3	40 (2.5 m^2^)	62.5	176 (11.0 m^2^)	56.8

Each time litter-soil samples with the total area of 0.5 m^2^ (8 samples with the total area of 1/16 m^2^) were collected from each site, except for site V.

Samples from site V, due to difficult field conditions (thickly overgrown with grass), were collected only twice (fall and winter).

Thus the overall number of 176 samples with the total area of 11 m^2^ was collected (Table 1).

Qualitative material from under coarse woody debris was also collected.

The collected material was handsorted. Specimens preserved in 75% ethanol are stored in the collection of the Department of General Zoology, Adam Mickiewicz University in Poznań. For the analyses, we applied standard methods and indicators used in faunistic research: number, population density, frequency, dominance, Morisita index as modified by Horn, the Shannon-Weaver diversity index, Pielou’s measure of species evenness. For species and site comparison, we applied the cluster method (nearest neighbor; distance/similarity measure – Bray and Curtis).

## Results

The research resulted in the collection of 608 specimens from 11 species of Chilopoda, nine from the order Lithobiomorpha and two from the order Geophilomorpha (Table 2).

**Table 2:** The number of specimens of individual Chilopoda species (in alphabetical order) at sites I-VI, and the frequency (F%), dominance (D%) (F and D calculated on the basis of quantitative samples), values of the Shannon-Weaver diversity index (*H*′), the highest values of the Shannon-Weaver index (*H*_max_) and Pielou’s measure of species evenness (*J*)

Species/ Site	I	D%	II	D%	III	D%	IV	D%	V	D%	VI	D%	I or II	Total	No. of sites	D%	F %
*Lithobius agilis* C.L. Koch, 1847	3	2.0	14	6.7							3	8.1		20	3	4.6	9.1
*Lithobius borealis* Meinert, 1868	16	10.5	3	1.5							6	16.2		25	3	5.7	8.5
*Lithobius curtipes* C.L. Koch, 1847	38	24.8	43	21.1	4	30.8	14	58.3	1	20.0	5	13.5		105	6	24.0	31.8
*Lithobius erythrocephalus* C.L. Koch, 1847											13	35.1		13	1	2.9	3.5
*Lithobius forficatus* (L., 1758)	22	14.4	13	6.4	1	7.7	1	4.2	2	40.0	4	10.8		43	6	9.8	11.4
*Lithobius lapidicola* Meinert, 1872	21	13.7	18	8.8			4	16.7			6	16.2		49	4	11.2	14.8
*Lithobius mutabilis* L. Koch, 1862	18	11.8	63	30.9	3	23.1	2	8.3					1	87	4	19.9	18.2
*Lithobius pelidnus* Haase, 1880	9	5.9	8	3.9										17	2	3.9	4.5
*Lithobius piceus* L. Koch, 1862	1	0.6	4	2.0									1	6	2	1.4	2.3
*Schendyla nemorensis* (C.L. Koch, 1837)			4	2.0			2	8.3	2	40.0				8	3	1.8	4.0
*Strigamia acuminata* (Leach, 1814)	25	16.3	34	16.7	5	38.5	1	4.2						65	4	14.8	18.2
*Lithobius* sp*.*	43		90		7		4		2		24			170			
Total number of specimens	196		294		20		28		7		61		2	608			
Total number of species	9		10		4		6		3		6						
Density (ind./0.5 m^2^)	49.0		73.5		5.0		7.0		2.3		15.3						
*H′*	0.85		0.83		0.55		0.56		0.46		0.73			0.91			
*H max*	0.95		1.0		0.6		0.78		0.48		0.78			1.04			
*J*	89.11		82.8		91.31		72.11		96.02		93.33			87.0			

The most numerous and most frequently found species in the community were *Lithobius curtipes*, *L. mutabilis* and *Strigamia acuminata*, respectively. *Lithobius piceus* and *Schendyla nemorensis* were the rarest and the least numerous species (Table 2).

All of the sites investigated featured two species – *L. curtipes* and *L. forficatus*.

Control sites showed the highest values for indices of species richness and diversity (Table 2). Only *L. erythrocephalus* was not found at these sites. It is in these areas exclusively that *L. piceus* and *L. pelidnus* were found. The density of centipedes was also the highest there (49.0 and 73.5 ind./0.5 m^2^, respectively).

The best living conditions for Chilopoda among the areas affected by the windstorm were offered by site VI – almost deprived of live trees but abundant in coarse woody debris. In total, six species were found there (Table 2). It is only in this area that *L. erythrocephalus* was captured. The Shannon-Weaver diversity index (*H′*), and the density of centipedes were the highest there in comparison with other damaged sites.

The least favourable conditions for Chilopoda were offered by site V. Only 7 specimens from three species were captured there. At this site, due to the fact that it was thickly overgrown with tall grass, half as many samples as at the other sites were collected.

The density of centipedes at the damaged site was very low and ranged from 2.3 to 15.3 ind./0.5 m^2^.

**Fig. 3:**
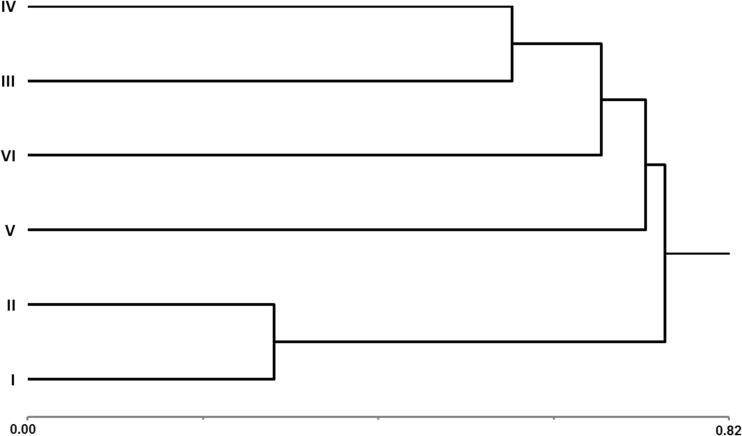
Site similarity. Distance/similarity measure – Bray and Curtis; cluster method – nearest neighbor

The similarity of individual sites in terms of the number and species composition of Chilopoda is shown in Fig. 3. Control sites (I and II) form a separate cluster. The most similar sites in that group of areas affected by the windstorm are sites with natural (III) and human (IV) planting of beech trees. The other two sites discussed above (V and VI) are the most distinct.

Similar patterns of occurrence are shown by the following species:

*L. curtipes* and *L. forficatus* – present at all sites, *S. acuminata* and *L. mutabilis* – reported from undamaged and regenerating sites, *L. agilis* and *L. borealis* – found at control sites and at the damaged site VI, as well as *L. pelidnus* and *L. piceus* captured only at the control sites (Table 2).

**Fig. 4:**
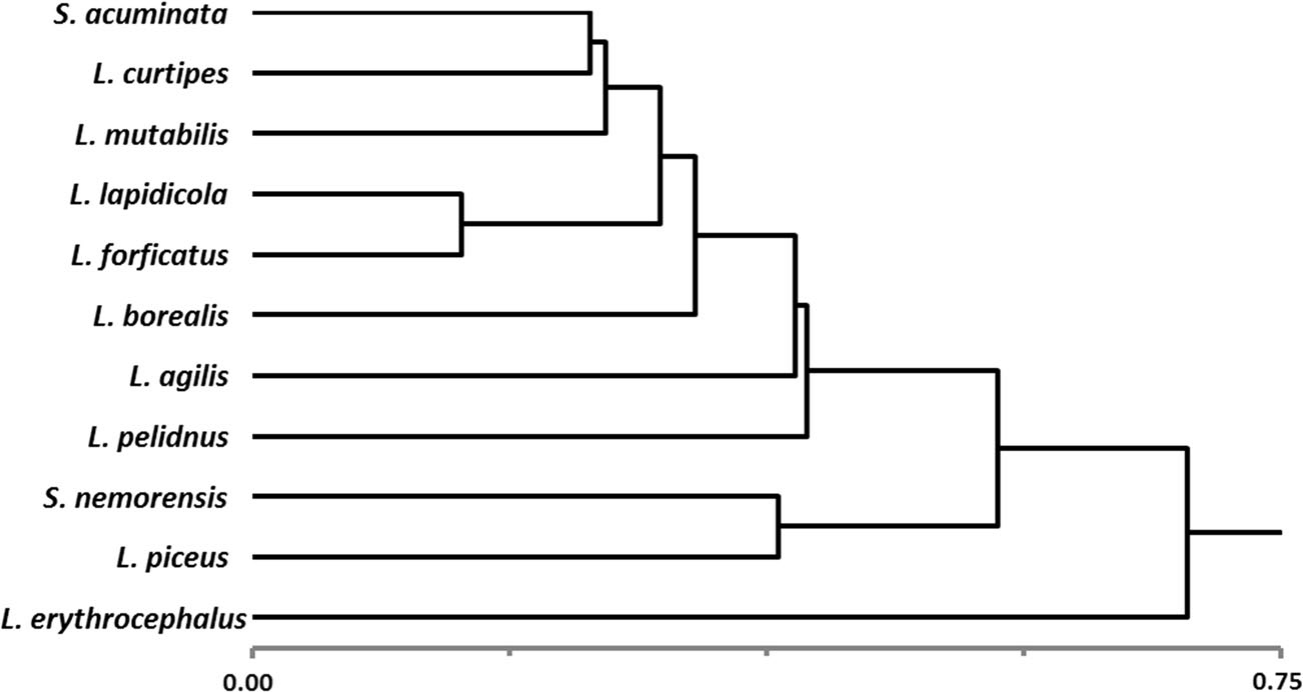
Species similarity. Distance/similarity measure Bray and Curtis, cluster method – nearest neighbor

Similarities between species (in terms of the number at individual sites) determined using the hierarchical clustering method are shown in Fig. 4. The distinct character of *L. erythrocephalus* is clearly evident. The most similar species include *L. forficatus* and *L. lapidicola* – featuring a similar number, and also *S. acuminata*, *L. curtipes*, and *L. mutabilis* – leading in the community.

Dominance structure (Table 2): Centipedes at control sites (I and II) show the most balanced, most complete dominance structures. No superdominants were found at site I (D > 30%), while *L. lapidicola* and *L. borealis*, apart from the most frequent and most numerous species in the reserve (*L. curtipes*, *S. acuminata*, *L. forficatus* and *L. mutabilis*), were also found among eudominants (D = 10.1–30.0%). The dominant (D = 5.1–10%) at this site was *L. pelidnus*, while the other two species have attained the status of a recedent (D = 1.1–2.0%) there – *L. agilis* or a subrecedent (D < 1.0%) – *L. piceus*.

*L. mutabilis* has attained the status of a superdominant at the control site II, whereas *L. curtipes* and *S. acuminata* – the status of eudominants. The group of dominants includes *L. lapidicola*, *L. agilis* and *L. forficatus*. *L. pelidnus* was a subdominant (D = 2.1–5.0%), while the other species were recedents.

Dominance structures were less balanced and shorter at sites damaged by the windstorm.

Two species at site III, *S. acuminata* and *L. curtipes*, have attained the status of superdominants. *L. mutabilis* was a eudominant, and *L. forficatus* a dominant.

Site IV – the status of a superdominant, greatly exceeding the number of other species, has been attained by *L. curtipes* (58.3%). *L. lapidicola*, as a eudominant, ranked second. The other species can be classified as dominants (*L. mutabilis* and *S. nemorensis*) and subdominants – *L. forficatus* and *S. acuminata*.

At site V (with the lowest number of species) the status of superdominants has been attained by *S. nemorensis* and *L. forficatus*, while *L. curtipes* was a eudominant at this site.

*L. erythrocephalus* was a superdominant at site VI and it was reported only from this site. Eudominants included *L. borealis, L. lapidicola*, *L. curtipes* and *L. forficatus*, while *L. agilis* has attained the status of a dominant.

Four species have attained the status of eudominants in the general structure, and these were respectively: *L. curtipes, L. mutabilis, S. acuminata*, and *L. lapidicola*. The dominants include *L. forficatus* and *L. borealis*, while the group of subdominants included *L. agilis*, *L. erythrocephalus* and *L. pelidnus.* The other species are recedents – *L. piceus* and *S. nemorensis*.

Similarity of dominance structure was expressed by Morisita index values as modified by Horn (Table 3). The highest similarity is manifested by dominance structures at control sites. High similarity is also typical of dominance structures at sites III and II, III and I, as well as III and IV, and also IV and I, IV and II, as well as IV and III, VI and IV.

**Table 3:**
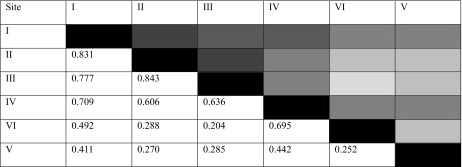
Similarity of dominance structures expressed by Morisita index values as modified by Horn (the darker the color the greater the similarity)

Site V is most distinct in terms of the dominance structure of all the sites and it shows the least similarity to any of the sites. However, the number of the collected specimens and species is exceptionally low there.

*L. curtipes*, *L. mutabilis*, *S. acuminata*, and *L. lapidicola* have attained the highest frequency.

The share of samples with centipedes was the highest at control sites (81.3 and 90.6%) (Table 1). It was also relatively high at sites VI (59.4%) and IV (40.6%), and the lowest at sites V and III.

Species diversity expressed by the Shannon-Weaver index (*H*′) reached the highest value at control sites and at site VI (Table 2).

## Discussion

The “Buczyna” reserve had never been previously studied in terms of Chilopoda. Thus we were unable to compare the present results with the previous ones.

The species composition of the centipede community in the reserve under investigation is similar to communities from other beech forests and also Polish oak-hornbeam forests (e.g. Leśniewska 1997, 2000; Leśniewska and Taborska 2003; Leśniewska et al. 2005) and Central European ones (e.g. Albert 1982; Fründ 1987; Spelda 1999).

A relatively low number of species from the order Geophilomorpha were found in the beech forest under investigation. Extending the study period and expanding the methods to include deep soil samples would probably result in finding other species of this order.

The species dominant at the majority of sites – *L. curtipes*, *L. mutabilis*, and *S. acuminata*, are often found among dominants in other European forests investigated in this respect (e.g. Albert 1982; Fründ 1987; Poser 1988; Leśniewska 1997; Spelda 1999).

As expected, the greatest species richness and diversity, as well as the highest densities of Chilopoda were reported from fragments of the beech forest that were not damaged by the windstorm, especially at site II with the largest share of coarse woody debris, and numerous lying tree logs in particular. In addition, control sites featured the most balanced dominance structures. The balance of the dominance structures shows maturity of a community, the equilibrium attained in such a community, and the greatest interspecies competition (Leśniewska 1997).

Centipedes in the area affected by the windstorm found the best living conditions at site (VI), which although almost completely deprived of trees was thickly covered by dead logs, branches and stumps. In our opinion, it is this very large amount of coarse woody debris that affected the result we obtained. The correlation between the amount of coarse woody debris and the richness of the soil fauna, including centipedes, has long been known (e.g. Samuelsson et al. 1994; Jabin et al. 2004, 2007; Hanula et al. 2006; Leśniewska and Leśniewski 2016). A large amount of woody debris increases the heterogeneity of the environment. Additionally, larger amounts of litter accumulate around coarse woody debris. Thus microhabitats which are favorable for centipedes (dependent on the high level of moisture due to the structure of integument) with different nutritional needs are formed. As is commonly known, though being nutritional generalists, Lithobiomorpha however prefer Collembola (feeding on fungi growing in damp litter and coarse woody debris) as well as larvae of dipterans, of other insects, and soft-bodied invertebrates, such as nematodes, annelids, slugs, spiders, juveniles, or recently moulted woodlice (Voigtländer 2011; Etizinger et al. 2013). Geophilomorpha attack small annelids, larvae of dipterans and other insects, snails, arachnids, millipedes, woodlice, thysanurans, diplurans and various pterygote insects (Voigtländer 2011). The thicker the litter layer and the greater the amount of dead wood in all its forms, the better the conditions for all of the aforementioned groups of animals. Additionally, this promotes the development of a richer community of Chilopoda in all respects.

It is understandable that species richness, diversity and densities of Chilopoda were all significantly depleted in areas affected by the windstorm, featuring an altered structure. Habitats transformed this way failed to offer living conditions for typical forest species – such as *L. pelidnus* and *L. piceus*. It is also understandable that all of the investigated sites were inhabited, although in small numbers and rarely, by a eurotypic *L. forficatus* and a forest species *L. curtipes*. The former species demonstrates an enormous spectrum of the occupied habitats, whereas the latter one, of a small size, occupies a deeper layer of litter, at the borderline with soil. Evidently, even transformed habitats in the forest under investigation provide the latter species with minimum living conditions. Additionally, we know that *L.forficatus* is a large and active species, with higher tolerance of low humidity (e.g. Lewis 1981). It seems able to move quickly into suitable habitats and microsites. *L.curtipes,* on the basis of its European distribution (N. to the White Sea area – e.g. Zenkova 2016) seems able to tolerate fairly extreme conditions. However, it cannot be ruled out that the species captured at the damaged sites were only visitors from the neighboring forest stands.

What is interesting and hard to explain is the occurrence of *L. erythrocephalus* exclusively at the site affected by the windstorm, deprived of live trees although rich in coarse woody debris (site VI). *L. erythrocephalus* is a eurotypic species, one of the most common ones in Poland (Kaczmarek 1979, 1980). It is probably relatively rare at other sites and due to this fact it was not captured during our one-year study. According to Voigtländer (2005), *L. erythrocephalus* is one of the inhabitants of dry and very dry habitats without relations to vegetation cover.

Regardless of the fact that leaving coarse woody debris is of significance for increasing the species diversity of Chilopoda, it should be borne in mind that due to the absence of canopy the moisture conditions, which are so important for the animals investigated, have significantly deteriorated. Indeed, the litter that is left is of significant thickness, yet it is dried up. This results in the qualitative and quantitative decrease of Chilopoda, and naturally of other soil animals, which centipedes feed on (Coyle et al. 2017).

Although a large area of the forest has not been damaged and it is directly adjacent to the altered parts, one cannot be certain that the Chilopoda community will return to its original state along with the regenerating forest. It happens occasionally that foreign species enter forests planted by humans. These species alter the original, natural structure of the centipede community. This is what happened, for instance, in the case of the oak-hornbeam forest in Greater Poland (W Poland) (Leśniewska et al. 2005), where *Lithobius microps* was introduced into the regenerating community. This species should be considered as a foreign element in our forests and a sign of some degeneration of the community. Thus far, in the course of our research, we have not yet reported such species which could contribute to an adverse degenerative change in the community of Chilopoda studied here at the Buczyna reserve.

Windstorms are natural phenomena in nature. The decline of a forest in this way provides an opportunity for new generations of trees to grow. It initiates succession phenomena, which may consequently lead to increased biodiversity. However, it seems to cause significant depletion of the soil fauna, including Chilopoda, for many years to come.
